# Development of a Systems Medicine Approach to Spinal Cord Injury

**DOI:** 10.1089/neu.2023.0024

**Published:** 2023-08-23

**Authors:** James D. Guest, Margot Kelly-Hedrick, Theresa Williamson, Christine Park, Daniyal Mansoor Ali, Ahilan Sivaganesan, Chris J. Neal, Charles H. Tator, Michael G. Fehlings

**Affiliations:** ^1^ Neurological Surgery and the Miami Project to Cure Paralysis, University of Miami, Miami, Florida, USA.; ^2^ Department of Neurosurgery, Duke University, Durham, North Carolina, USA.; ^3^ Massachusetts General Neurosurgery, Harvard University, Boston, Massachusetts, USA.; ^4^ Department of Neurosurgery, Thomas Jefferson University, Philadelphia, Pennsylvania, USA.; ^5^ Division of Neurosurgery, Walter Reed National Military Medical Center, Bethesda, Maryland, USA.; ^6^ Division of Neurosurgery and Spine Program, Department of Surgery, University of Toronto, Toronto, Ontario, Canada.

**Keywords:** allostatic, biomarker, homeostasis, prognosis, spinal cord injury, systems biology

## Abstract

Traumatic spinal cord injury (SCI) causes a sudden onset multi-system disease, permanently altering homeostasis with multiple complications. Consequences include aberrant neuronal circuits, multiple organ system dysfunctions, and chronic phenotypes such as neuropathic pain and metabolic syndrome. Reductionist approaches are used to classify SCI patients based on residual neurological function. Still, recovery varies due to interacting variables, including individual biology, comorbidities, complications, therapeutic side effects, and socioeconomic influences for which data integration methods are lacking. Infections, pressure sores, and heterotopic ossification are known recovery modifiers. However, the molecular pathobiology of the disease-modifying factors altering the neurological recovery-chronic syndrome trajectory is mainly unknown, with significant data gaps between intensive early treatment and chronic phases. Changes in organ function such as gut dysbiosis, adrenal dysregulation, fatty liver, muscle loss, and autonomic dysregulation disrupt homeostasis, generating progression-driving allostatic load. Interactions between interdependent systems produce emergent effects, such as resilience, that preclude single mechanism interpretations. Due to many interacting variables in individuals, substantiating the effects of treatments to improve neurological outcomes is difficult. Acute injury outcome predictors, including blood and cerebrospinal fluid biomarkers, neuroimaging signal changes, and autonomic system abnormalities, often do not predict chronic SCI syndrome phenotypes. In systems medicine, network analysis of bioinformatics data is used to derive molecular control modules. To better understand the evolution from acute SCI to chronic SCI multi-system states, we propose a topological phenotype framework integrating bioinformatics, physiological data, and allostatic load tested against accepted established recovery metrics. This form of correlational phenotyping may reveal critical nodal points for intervention to improve recovery trajectories. This study examines the limitations of current classifications of SCI and how these can evolve through systems medicine.

## Current Lack of Integration of Multi-System Effects of Spinal Cord Injury

*Symptoms, then are in reality nothing but the cry from suffering organs*.—Jean-Martin Charcot, 1868

*Science may be described as the art of systematic oversimplification—the art of discerning what we may with advantage omit.—*Karl Popper, 1982^1^

**FIG. 1.**
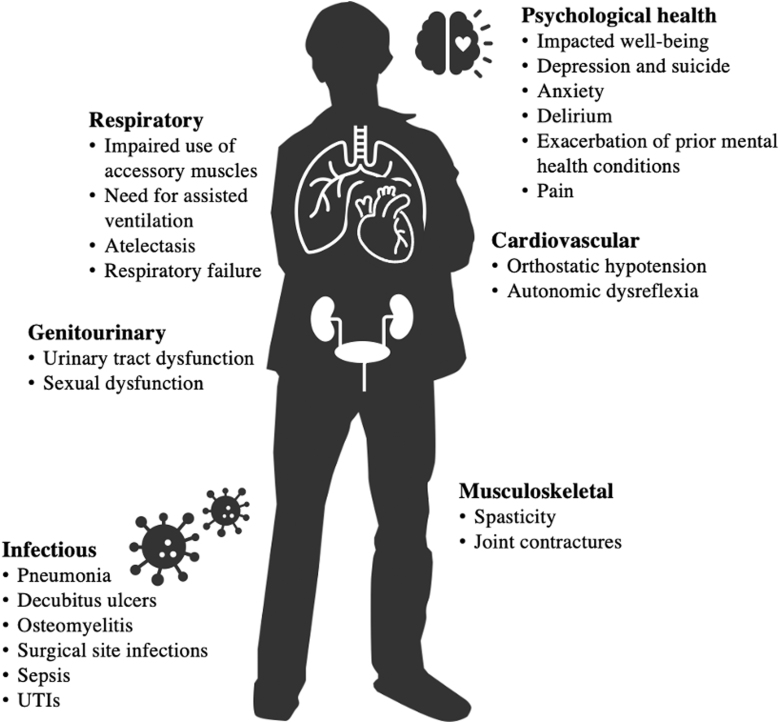
Spinal cord injury is a multi-system disease. System changes are inter-dependent.

Traumatic spinal cord injury (SCI) is one of the most complex medical conditions, an acute event followed by both recovery and chronic disease. Severe cervical SCI impacts nearly all bodily organ systems. This is reflected in SCI medicine textbooks, where each system requires a separate expert chapter. However, integrated models of how the altered systems interact after injury are lacking (Fig. 1). Normally, the spinal cord is instrumental in the homeostatic feedback circuits of multiple systems. The acute injury abruptly disrupts these systems that evolve into different functional states with differing time courses across individuals. Thus, we observe that the severity of neuropathic pain, dysautonomia, immunological dysfunction, spasticity, and age-accelerating states like metabolic syndrome vary among people with the same initial injury pattern. Data gaps between the acute, subacute, and chronic phases also impede detecting key events that drive eventual phenotypes. Although we often speak of the post-injury phase as recovery, on a systems level, it primarily consists of adaptations to the loss of prior homeostatic feedback controls, some of which are maladaptive. Previously, most acute research has focused on the injury site, and numerous localized molecular effects limiting recovery were identified in experimental models.^2,3^ However, as the importance of multi-system-distributed changes becomes increasingly evident,^4^ a broader perspective is required.

Precision medicine seeks to tailor care to individual patients based on unique features. For SCI, this will require the ability to define subtypes from several classes of relevant data (e.g., clinical, imaging, molecular, physiological) that capture evolution to different chronic SCI states. Using examples from traumatic brain injury,^5^ we describe methods to classify SCI syndromically according to multi-variate phenotypes deduced from similarity analysis clusters.^6^ These analyses may uncover common shared signaling modules and critical transitional events that underlie secondary complications by integrating phenotypic and molecular networks that drive pathophysiologies.^7^

**FIG. 2.**
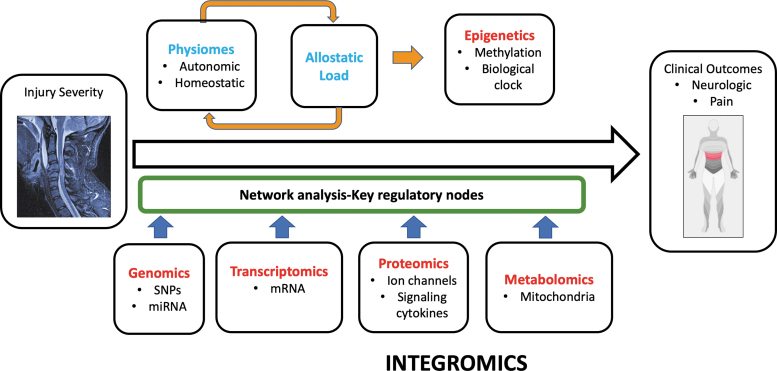
Integromics: Spinal cord injury disrupts numerous homeostatic feedback circuits. Initial injury severity is the baseline variable from which a variety of outcomes may occur including secondary phenotypes such as neuropathic pain. Critical transitions underlay the evolution of these phenotypes to be explored against bioinformatics data: Genomics, Transcriptomics, Proteomics, Metabolomics: Epigenetic change is anticipated to be driven by allostatic load. Bioinformatic and clinical data is integrated in a network analysis to identify the most highly correlated factors influencing phenotypes. This conceptual figure is based on Figure 1 from Morris and Baladandayuthapani.^388^

Systems medicine is dedicated to deciphering diseases at the comprehensive individual level, revolving around the idea that specific phenotypes reflect complex, multi-layered molecular and physiological interactions. Both intrinsic and extrinsic factors influence dynamic post-SCI inter-system interactions. An “integromics” approach to SCI quantifies interactions between discrete organ system disturbances within physiological and molecular networks^4,8–12^ “Syndromics” intends to generate a consolidated picture of an affected individual by integrating mechanistic biological data with clinical measures.^12^ Integrative physiology and systems biology^13^ are co-evolving disciplines to quantify disease-induced disruption in interconnected biological networks that bridge traditionally separate systems (Fig. 2).^14^ This approach underlies the concept of emergence, wherein complex biological systems exhibit composite higher-level properties derived from the interactions of components.^15^ Attributes such as resilience, frailty, and aging are emergent properties relevant to SCI.

Systems biology has been used to develop integrative models^16^ in multiple sclerosis (MS)^17^ and amyotrophic lateral sclerosis (ALS).^18^ Normally, the spinal cord serves as a multi-organ physiological adaptive, and stabilizing network. SCI disruption drives evolution to new dysregulated states seen as phenotypes such as neuropathic pain and dysautonomia based on neurophysiological and molecular changes. To model a phenotypic evolution framework for SCI, we propose combining physiological knowledge with network analysis from systems biology,^19^ allostatic stress indices,^13^ and Physiome computational biology models.^20,21^

Current SCI classification systems are based on aggregated natural history observations derived from periodic neurologic physical examinations^22^ and outcome measures assessing function and self-care^23^ that result from combinations of neurological recovery and adaptive strategies. There is a lack of methods to integrate chronic secondary conditions such as neuropathic pain,^24^ autonomic dysfunction,^25^ spasticity, muscle atrophy and deconditioning,^26^ bone mass loss,^27^ immune dysfunctions,^28^ chronic inflammation,^29^ and metabolic syndrome (Table 1).^30^ Acute complications, such as infections that can negatively impact the initially predicted outcomes, are called “disease-modifying events.”^25,31-33^ However, the mechanisms by which these events influence the evolving neural injury remain unclear. Some systemic biomarkers have been mechanistically linked to complication states. For example, reduced leukocyte human leukocyte antigen-DR (HLA-DR) levels can indicate post-SCI immune depression syndrome, increasing vulnerability to infections.^34^

**Table 1.** Multi-System Effects of SCI

Multi-system effects of spinal cord injury*^364^*	
Immune	Post-SCI immune dysfunction syndrome and autoimmunity.^29^ Elevated inflammatory cytokines.^365^
Cardiovascular	Debilitating orthostatic hypotension both in the acute and chronic phases. Dysregulated sympathetic activity, termed autonomic dysreflexia. Cardiovascular diseases are the second most common cause of death of individuals with traumatic SCI in the long term.^130^ Deep venous thrombosis and pulmonary embolism.
Respiratory	Respiratory complications account for nearly one-third of deaths in the year after SCI and continue to be high throughout life.^366^ Cervical and upper thoracic injuries often impair respiratory function.^367,368^
Renal	UTIs, renal calculi, renal failure.^369^
Intestinal	Complex changes to intestinal health follow SCI^370^ and changes in gut microflora influence recovery.^371^
Skin	Vulnerable to pressure ulceration and infection. In the acute phase, 30-40% of patients will develop a pressure ulcer, significantly impacting their ability to recover and participate in rehabilitation.^372^
Bone	Bones mass loss eventually creates fracture risk.^27,373^ Heterotopic ossification can cause pain and stiffness.^374^
Muscle	Muscles undergo changes in fiber type, fibrosis^26^ and may lose their endocrine functions.^341,375,376^
Metabolic	Impaired glucose tolerance and diabetes risk. Increases in body fat contribute to proinflammatory metabolic syndrome
Endocrine	Reduced growth hormone and testosterone.^377,378^ Hypothalamic-pituitary–gonadal axis.^379^
Psychological	Increased rates of anxiety and depression,^380,381^ substance abuse, impact of resilience^380,382^

SCI, spinal cord injury; UTI, urinary tract infection.

Homeostatic processes, such as metabolism, require physiological integration across organ systems.^35^ Normally, spinal cord integration of physiological systems operates at multiple levels of complexity. SCI disrupts these interdependent, feedback-regulated systems with some effects dominated by the rostral-caudal level where neural axis injury occurs,^4^ and others correlated with injury completeness.^36^ A systems approach to understanding, treating, and mitigating the consequences of SCI involves several hierarchical levels. Examples include the molecular pathobiology of inflammatory cells,^37^ epigenetic changes contributing to neuropathic pain,^38^ gut population dysbiosis, physiological variables such as muscle tone and blood pressure,^39^ and system-level impacts on metabolism, inflammation, and immune function.

How do we move from the present state of SCI knowledge toward more integrated models? Here, we review existing prognostic and classification methods and suggest working backward from clustered phenotype combinations to learn the critical events in their evolution. We suggest these “complication” phenotypes involve transitions in “state” away from homeostasis in the molecular-physiological systems that will have consistent underlying topologies.^40,41^ We propose testing the evolving phenotypes against multi-variate data to derive the most significant physiological and molecular alterations. If such a phenotypic-bioinformatics framework is established, integration of evolving individual injury data may enable the prediction of eventual neurological and complication phenotypes,^42^ health outcomes,^43^ and better inform treatment interventions. The steps include identifying molecular fingerprints for phenotypes as molecular signals rising above the noise level and their conceptualization within mechanistic computational models.^44^

**FIG. 3.**
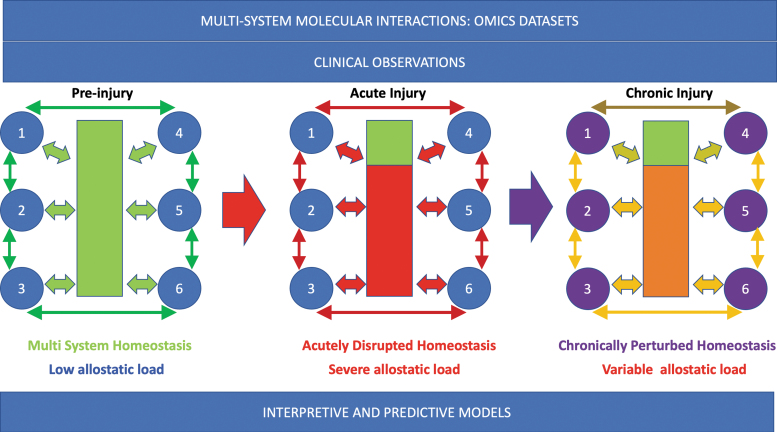
The effect of spinal cord injury on multi-system homeostasis. In the uninjured system, the spinal cord (green box) integrates feedback systems (e.g., 1-6) between multiple levels of the spinal cord and organ systems. Although stresses occur, the state returns to baseline. During acute injury, homeostatic circuits are severely interrupted (red box, below injury level). This generates severe allostatic load. In the chronic injury phase, the homeostatic disruption persists. Some systems may have intermittent severe allostatic load, such as when autonomic dysreflexia occurs. For autonomic dysfunction, the level of injury and severity are important determinants of disruption severity.

**FIG. 4.**
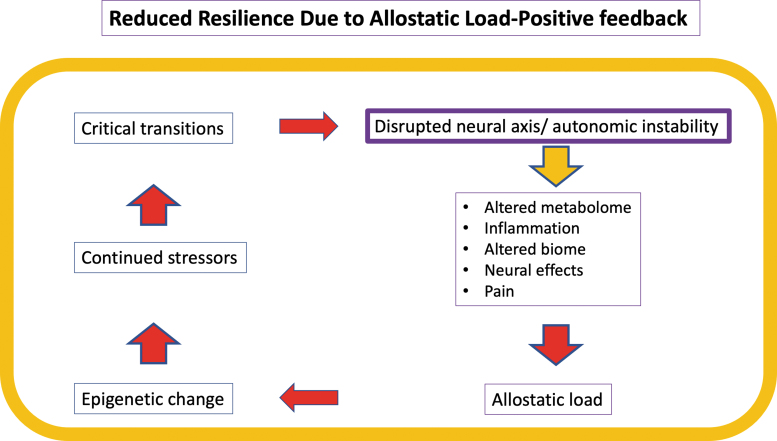
Allostatic load positive feedback. Loss of normal feedback functions after spinal cord injury (SCI) perpetuates and accelerates reduction in the emergent property-resilience. High resilience counters allostatic stress. Here, allostatic load drives epigenetic change, which in the face of continued stressors produces the critical transitions underlying chronic SCI phenotypes such as neuropathic pain, metabolic syndrome, and spasticity.

Normal network physiology requires rapid stabilizing feedback,^45,46^ regulated mainly by the central and peripheral nervous and neuroendocrine systems. Allostasis refers to the dynamic regulatory adaptations within normal physiological ranges that maintain stability in physiologic systems such as autonomic, central nervous, neuroendocrine, cardiovascular, metabolic, and immune systems.^47,48^ Allostatic mechanisms restore “homeostasis” after perturbations through feedback loop corrections, including thermoregulation, peripheral vascular resistance, and inflammation control. Following SCI, the adaptive capacity of allostatic feedback is variably lost, mainly due to distributed autonomic system disruptions (Fig. 3).^49^ When a physiological system is destabilized beyond homeostatic boundaries, allostatic load (AL) is generated. Chronic AL contributes to accumulating damage, reduced resilience, accelerated system aging,^50^ and potentiation of adverse health conditions.^51^ After SCI, many systems cannot “normalize,” resulting in measurable AL (Fig. 4),^52^ increasing vulnerability to metabolic syndrome, cardiovascular disease (CVD), infection, chronic pain,^53^ and chronic inflammation due to neuroendocrine and immune dysfunction.^28^ Overall, these changes may reduce resilience and increase frailty.^54^ At a genetic level, polymorphisms may increase vulnerability to developing AL,^55^ and AL can drive epigenetic change within the individual genome.^56^

Systems biology examines perturbations of pathway kinetics,^57^ primarily using omics data sources. Computational and statistical tools identify connections across prominent molecular nodes and modules using machine learning (ML), dimensionality reduction, and network analysis,^58,59^ A major challenge is understanding the linkage between molecular events and physiology. Physiomes are quantitative physiological models that incorporate data from relevant biological scales to create models useful in real-time.^20^ Physiomes altered by disease-related changes are “pathomes.”^60^ A cardiovascular Physiome has been applied to traumatic burn injuries,^61^ and there is progress toward constructing brain physiomes.^62^ By adjusting variables in Physiome models, researchers can rapidly predict changes within the system. Physiome approaches are considered “top-down” modeling approaches, whereas systems biology is a “bottom-up” data-driven approach. Physiomes involve describing physiology with mathematical equations that are necessarily oversimplifications. There is a tension in modeling between high granularity and fidelity versus the need for simplifications to achieve utility for timely application.^63^ An allostatic load Physiome may inform how homeostatic circuits dysregulated after SCI switch from negative feedback to positive feedback, potentiating and maintaining secondary phenotype states.

Thus, we propose incorporating AL as a key progression driver in our multi-variate approach to the evolution and maintenance of chronic SCI phenotypes, reduced resilience, and system aging. If this hypothesis proves correct, reducing AL could become a therapeutic goal to mitigate accelerated aging and complications in chronic SCI^64^ through interventions such as exercise, drugs (anti-depressant/pain), and other treatments.^65,66^

### Phenotypes, syndromes, states, and transitions

We propose to use “phenotype” for a definable post-SCI secondary condition such as neuropathic pain, metabolic syndrome, spasticity, and dysautonomia. Identifying critical transitional “states” during post-SCI evolution may be possible through molecular bioinformatics to identify “tipping” points that establish these phenotypes. We further hypothesize that syndromic modules of gene expression, epigenetic change, allostatic load, and physiology will underly these phenotypes and may be opportunities for therapeutic intervention.

This article begins by examining conventional classifications, outcome measures, and established prognostic indicators for SCI, including early magnetic resonance imaging (MRI) structural and serum/cerebrospinal fluid (CSF) biomarkers. We then address the challenges of reconciling clinical and systems biology terminology and integrating current clinical measurements with molecular network analysis into statistical models. Finally, we propose an integrated multi-systems conception of SCI extending from the acute injury phase through the subacute and into the chronic disease phase. In doing so, we begin to bridge the wealth of knowledge regarding acute injury in the domains of SCI critical care with those of rehabilitation, physiology, and bioinformatics science.

## Current SCI Classification

*The word is not the thing, the map is not the territory*—from the Meaning of Meaning. Ogden and Richards, 1923^67^

The classification of a medical problem greatly affects how we think about it. Medical classification methods emerged from the clinical necessities of diagnosis, prognosis, and treatment selection. For daily practice, medical practitioners choose classification methods that are reliable, straightforward, cost-efficient, and broadly accepted. Historically, classification was based on clinically pertinent physical examination and symptom distinctions that described the observable disease phenotype. In contemporary medicine, there is increasing emphasis on mechanistic molecular pathobiology in cancer,^68^ inflammatory,^69,70^ and autoimmune conditions^71^ as more specific determinants for classification, prognosis, and treatment. Alignment to molecular pathobiology quantitatively and temporally during disease phases serves as a rational basis for therapeutics discovery and development.^72,73^ Bioinformatics has enabled unprecedented systemic and organ-level insights into interacting gene transcription networks.^74^ Genomics and transcriptomics have revealed that individual heterogeneity is normative in neurological diseases^75-77^ even when external phenotypes appear similar. Further, the observed heterogeneity of therapeutic efficacy to improve recovery after SCI suggests that interindividual variation and multi-system complexity are underlying factors. Consequently, new modeling and classification methods are needed.^78^

### Prediction versus explanation

In the months after SCI, both recovery and multi-system adverse changes simultaneously co-occur, with some interposed complications worsening the recovery trajectory.^32,33^ Eventually, recovery slows down, but many system changes continue to evolve, creating uniquely individual chronic SCI states. Changes, such as loss of bone mass, altered gut microbiomes, and increased visceral body fat content, are not obvious. Current SCI classifiers (Supplementary Table S1), such as the American Spinal Injury Association (ASIA) Impairment Scale (AIS),^22^ encompass broad states that greatly over-reduce the complexity of SCI (Supplementary Fig. S1). Although early damage biomarkers such as released structural proteins and MRI changes^79^ may correlate with current SCI classifiers, significant physiological systems are not incorporated, and the markers do not reveal the underlying causative molecular changes.

Reductionist approaches are essentially blind to integrated systems effects, with limited predictive power for eventual functional recovery, complications, and health states.^19^ Current SCI predictive models primarily focusing on motor and sensory recovery are most accurate at the extremes of injury (worst and least severe). Reductionist approaches do not encompass emergent network properties impacting recovery, such as resilience.^80^

### SCI classifications that are also outcome measures

Optimal outcome measurements are relevant throughout a disease population and usable by many healthcare practitioners with strong metric properties.^81,82^ Typically, after an SCI, there is immediate maximal functional loss followed by some recovery, primarily in the first year.^83^ Existing SCI classification frameworks, which capture changes in neurologic function and independence during recovery, are based on identifying the injury level and residual function within the body's myotomes and dermatomes. The neurological classifications AIS and International Standards for Neurological Classification of Spinal Cord Injury (ISNCSCI) have enabled valid longitudinal comparisons for research, including therapeutics.^84,85^ Following SCI, the ability to live independently correlates to measurement scales of function, including the Spinal Cord Independence Measure (SCIM),^23^ which originated from the Functional Independence Measure (FIM).^86,87^ In the current prognostic framework, a common dependent variable is a measure of disability correlated to independence and care needs.^23^ SCIM-III does not, however, provide information about chronic complication phenotypes.

The International Spinal Cord Society and the American Spinal Injury Association (ASIA) collaborated to create the ISNCSCI,^22^ which has evolved over decades of refinement. Standardized examination procedures are used to determine the AIS, a single ordinal severity score, and the ISNCSCI ordinal motor and sensory test scores are often treated as interval measures.^88^ The primary classification tool is the ISNCSCI score sheet A. Data are entered, summarized, and checked according to online algorithms to define the neurological level of injury (NLI), AIS (incompleteness status), and motor and sensory preservation patterns.^89^ This visual mapping and numerical methodology form the current nosology of SCI, allowing the extraction of SCI patterns such as central cord injury.

The widespread international adoption of ISNCSCI facilitates consistent communication and comparison among rehabilitation institutes, research studies,^90^ and across languages.^91^ The assessments are low-cost and practical to describe changes in the neurological preservation map over time.^92^ However, the ISNCSCI requires significant training and incorporation of updates, is time-consuming is subject to classification errors,^93^ is insensitive to subtle neurological recovery,^94^ and does not always align with function. An “expedited” version has been developed to shorten the assessment duration for initial screening examinations.^95^ Standards of autonomic assessment have also been established to provide standardized assessments for residual sympathetic and parasympathetic function.^96^

Sources of classification heterogeneity affecting ISNCSCI include a lack of incorporation of non-neurologic injuries such as limb fractures and nerve and muscle injuries, which can reduce motor and sensory scores not directly attributable to the SCI and exhibit different recovery profiles. To address this issue, the ISNCSCI has added an asterisk to the recorded score,^97^ a practical but abstract qualifier.

In up to 80% of patients, traumatic SCI results in multi-system injuries whose long-term impact is poorly understood. Acute systemic trauma measures, the Abbreviated Injury Score, the Injury Severity Score (ISS), and the Acute Physiologic Assessment and Chronic Health Evaluation II (APACHE II) score are used for mortality prediction.^98–101^ Multiple organ dysfunctions are frequent during intensive care after traumatic SCI.^102^ The Spinal Cord Injury Risk Score, specifically designed to integrate overall trauma burden in acute SCI, demonstrated superior mortality prediction compared to ISS in a machine learning analysis.^103^ A multi-variate Fine-Gray cumulative risk model predicted the duration of intensive care unit (ICU) stay and mortality by incorporating neurological level, total motor score, and organ failure defined from laboratory values and ventilatory status at Day 4 post-SCI,^104^ demonstrating that ISNCSCI could be modeled together with critical care variables.

### Limitations of current classifications: Abstract reductionist systems

**FIG. 5.**
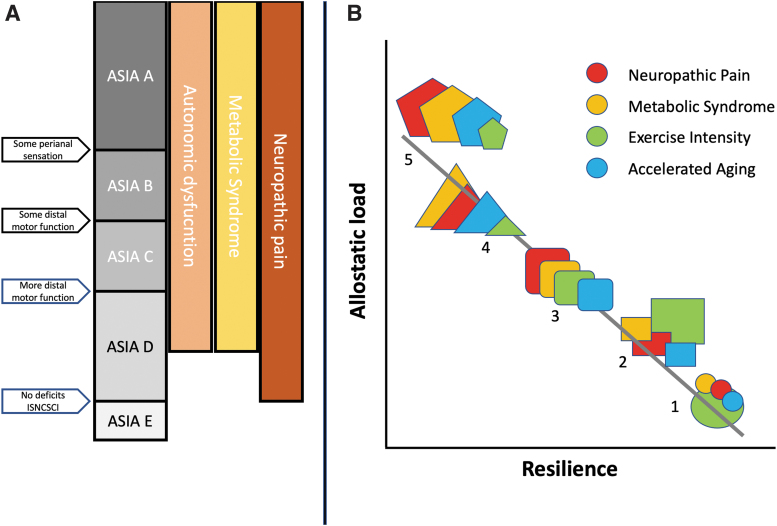
Syndromic classification of spinal cord injury. **(A)** American Spinal Injury Association (AISA) impairment grade scale is defined by only four distinctions, left arrows. Important syndromic effects of spinal cord injury span these grades, as illustrated for autonomic dysfunction, metabolic syndrome, and neuropathic pain. **(B)** Resilience is an important emergent factor in living with a spinal cord injury. Allostatic load may reduce resilience. Five possible individuals (shown by different shapes) are mapped to the Allostatic Load-Resilience Axes according to the severity (shown as size) of neuropathic pain, metabolic syndrome, accelerated aging, and the mitigating factor of intensive exercise.

Given the multitude of systems changes after SCI, a five-category AIS classification^22,105^ based on two criteria (motor/sensory) is overly reductionist. Significant consequences of SCI, such as neuropathic pain, spasticity, and dysautonomia, can span several AIS categories (Fig. 5A). AIS is sometimes used circularly, such as when it is assumed that AIS B status predicts conversion to AIS C. This relationship appears meaningful but provides almost no mechanistic information. Thus, the AIS and ISNCSCI are ordinal neurological severity scores useful to specify the neurological level and motor and sensory impairments, with limited correlation to changes in function^106,107^ unless combined with other clinical tests.^108^ Ordinal measures cannot provide the mechanistic insights of systems medicine approaches^109,110^ to capture the dynamic, multi-system disrupted state of SCI where several factors interact to influence recovery trajectories and chronic life with SCI.^111^ Major secondary complications such as neuropathic pain, heterotopic ossification, spasticity, and dysautonomia^112,113^ may be significantly disabling despite limited motor and sensory recovery. Due to familiarity, the ISNCSCI has greatly dominated as a SCI clinical trial outcome measure obscuring other outcomes of importance.

## Other Predictors of Patient Recovery after SCI

The post-SCI recovery phase, mainly the first year, is when most patient data has been acquired during acute and rehabilitation periods. Recovery prediction is dominated by SCI severity, rostral-caudal level in the neural axis,^114-116^ and age.^117,118^ Incomplete,^119,120^ more caudally located injuries with less severe MRI findings are associated with more motor recovery.^115^ Extrinsic sources of recovery trajectory variation include early transfer to Level 1 care,^121^ time to surgical decompression,^122^ and timing of rehabilitation.^121^ Longer-term external influences include contextual and motivational factors such as activity intensity and participation.^123^

Multi-variate models have been developed to predict functional outcomes based on current classifiers. Wilson and colleagues developed a model to predict independence at one year using linear and logistic regression.^116^ Bootstrapping was used to assess the robustness of the model.^116^ The model was simplified by dichotomizing total motor scores and converting AIS into a number.

A systematic review assessed Motor FIM (mFIM) recovery predictors using the International Classification of Functioning, Disability, and Health (ICF) domains of body structure and function, activity, participation, and context.^123^ The strongest positive predictor was rehabilitation duration, while older age and delayed admission to rehabilitation predicted less mFIM recovery. Vulnerability to depression and anxiety has also been linked to less favorable functional outcomes.^124-126^ Recovery is thus a broad concept with multiple intrinsic and extrinsic contributing factors.

### Predictors of mortality

Mortality rates after SCI have decreased over time,^127^ with high-level cervical injury and completeness as consistent predictors.^12^ Multi-system injuries, comorbidities, age, frailty, and concurrent traumatic brain injury correlate with higher in-hospital mortality rates.^54,128,129^ Chronically, cause-specific mortality is substantially higher in people with SCI than in the age-matched general population, with respiratory, cardiovascular, and urogenital problems being the leading causes of mortality.^130,131^

### Demographic predictors

Complex sociodemographic factors influence a patient's care and recovery. Demographic factors are not generally incorporated into SCI recovery models, but biological sex,^132,133^ race, and ethnicity^134,135^ influence neurological outcomes. Other sociodemographic factors, such as education, social support, language, insurance status, attitudes to disability, and re-employment^136^ impact treatment and recovery. To enhance predictive modeling, SCI researchers and clinicians should aim to incorporate race, ethnicity, and socioeconomic variables in their analyses.^137^

## Biomarker Modeling in SCI Prediction and Prognostication

Significant effort has been devoted to developing SCI biomarkers to achieve more individualized outcome predictions and to inform pathophysiology. Biomarkers are fundamental to precision medicine. Accurate outcome modeling is essential for designing clinical trials and interpreting therapeutic effects in the context of patient heterogeneity, especially in incomplete SCI (AIS B-D), where large standard deviations in outcome values are observed. This variability has necessitated enrolling large patient groups for treatment research, which can be impractical.^138^

Therapeutics development requires pharmacodynamic and surrogate endpoint response biomarkers^139^ to add an unbiased quantitative dimension to prediction algorithms based on AIS and ISNCSCI outcomes. As defined by the National Cancer Institute, biomarkers are “biological molecules found in blood, other body fluids, or tissues that serve as indicators of a normal or abnormal process, or of a disease or condition.” For SCI, structural, physiological, and molecular biomarkers are described.

To clarify the value of biomarkers for prediction and mechanistic pathobiology, it is essential to understand whether they are “indirect” descriptive injury markers or mechanistic components in disease pathogenesis.^140^ Some quantifiable disease biomarkers embedded in other disease molecular mechanisms are amyloid-beta and tau proteins, while others such as C-reactive protein (CRP), and hemoglobin A_1c_ are established disease surrogates. The Biomarkers, EndpointS, and other Tools (BEST) glossary was created by the U.S. Food and Drug Administration (FDA)-National Institutes of Health (NIH) Biomarker Working Group to promote consistency, clarity, and harmonization in the use of terminology related to biomarkers, endpoints, clinical research tools, and therapeutic product development.^141^ An important distinction is that between predictive and prognostic biomarkers.^142^

### Biomarkers of structural injury: Magnetic resonance imaging

MRI revolutionized our understanding of injury patterns and pathophysiology after SCI by directly visualizing injured tissue, influencing patient classification, treatment plans, and prognosis (Supplementary Table S2).^143^ MRI allows visualization of compression, edema extent, hemorrhage, and tissue disruption, which can be quantified.^144^ MRI has been used to predict neurological injury severity^145^ and outcomes^146–149^ with some structural markers correlating with recovery and secondary conditions in chronic phases.^149,150^ According to the BEST definitions, MRI can be a diagnostic, prognostic, monitoring, and safety biomarker in different contexts.

Injury severity, marked by intrinsic signal changes in acute T2-weighted MRIs, is most often correlated to clinical outcomes.^151^ The Brain and Spinal Injury Center (BASIC) MRI score, an ordinal scale describing five patterns of intramedullary T2 signal abnormalities in axial T2-weighted images, has been correlated with AIS scores during hospital admission and discharge.^152^ Other MRI features correlating with clinical outcomes include detectable intra-axial blood, linear edema extent, and spinal cord compression severity.^153,154^ Diffusion tensor imaging quantifies axonal pathway disruption, while magnetization transfer imaging signal changes have been associated with neurological function and outcomes.^155,156^ Currently, MRI is primarily used as a structural as opposed to mechanistic molecular biomarker but functional MRI, connectomics, spectroscopy, and integration with neurophysiology may reveal mechanistic changes related to the evolution of post-SCI phenotypic states such as neuropathic pain.^157^ Projects such as Enhancing Neuroimaging Genetics through Meta-Analysis (ENIGMA) correlate brain structure changes to regional gene expression.^158^

### Biomarkers from CSF and blood

After SCI, tissue damage and blood-brain barrier disruption cause cell content leakage into serum and CSF.^159^ Several serum and CSF biomarkers have been linked to neural tissue injury severity and neurological outcomes,^160^ with serial assessments capturing dynamic changes being more informative than single time-point cross-sectional analyses.^161,162^ Certain biomarkers have potential for patient stratification in clinical trials,^160,163^ monitoring therapeutic responses^139^ and indicating targets for new therapies.^164^

Fluid biomarkers studied for correlation to pathologic consequences include structural cytoskeletal,^165,166^ cytoplasmic cytokine signaling proteins, lipids, and microRNA (miRNA; Supplementary Table S3).^167,168^ Blood reflects systematic injury responses,^169^ including acute response and coagulation systems,^170^ whereas CSF is more directly linked to the injury region.

#### Cerebrospinal fluid biomarkers

Spinal CSF pressure monitoring in trauma protocols allows for serial CSF^159,160^ sample collection, enabling retrospective correlations between structural and inflammatory biomarker combinations, AIS grade conversion, and motor score recovery.^79,171^ One prognostic model using S100β, glial fibrillary acidic protein (GFAP), and IL-8 levels at 24 h post-injury predicted acute AIS grade with nearly 90% accuracy.^159^ Another model using IL-6, IL-8, MCP-1, tau, S100β, and GFAP predicted AIS grade conversion with 80% accuracy.

#### Serum biomarkers

Serum spinal cord structural injury biomarker concentrations are lower than in CSF but also have been correlated with probable AIS at different time-points after SCI. Elevated blood levels of GFAP, neuron-specific enolase (NSE), and phosphorylated heavy and light subunits of neurofilament (pNF-H/L)^172^ correlate to more severe acute traumatic SCI.^165,173^ NF-L levels, established as biomarkers in neurodegenerative diseases,^174^ were significantly associated with ASIA motor scores at baseline, 24 h, and 3 and 12 months post-injury.^165^ In North American Clinical Trials Network (NACTN) studies of riluzole neuroprotection, serum pNF-H levels have been correlated to an optimal neuroprotective dose.^139^ The protein degradome, reflecting the proteolytic activity of critical injury enzymes such as calpains and matrix metalloproteases, provides an index of their activity in injured tissue.^175,176^

### Inflammatory response biomarkers

Inflammation is a complex multi-system process that can be both beneficial and harmful. Increased inflammation is a major secondary effect of traumatic SCI with both extensive local injury and systemic inflammatory responses. Most complications following SCI have been linked to inflammation. Tumor necrosis factor alpha (TNF-α) and interleukin 1-beta (IL-1β)^177^ are important inflammatory signaling cytokines evaluated as predictive biomarkers. The inflammasome protein complex amplifies inflammation through caspase activation of IL- 1β^178^ and pyroptosis.^179^

Additionally, insulin-like-growth-factor 1 (IGF-1), transforming growth factor β (TGF-β), and soluble CD95 ligand (sCD95L) have been observed to increase following SCI. Patients with higher initial IGF-1 and sCD95L levels showed no improvement at 3 months post-SCI, while elevated IGF-1 was associated with neurological recovery in another study.^161,180^ Elevated TNF-α has been associated with both the development of the neuropathic pain syndrome^181^ and recovery probability.^177,182^ Merely measuring the quantity of these biomarkers is insufficient; a comprehensive framework that correlates the levels with patient outcomes is necessary for meaningful interpretation. Quantitative biological approaches are strengthened when a biomarker quantity is continuously linked to disease severity, such as NF levels in neurodegenerative disease.^174^

### Peripheral blood test laboratory biomarkers

Peripheral blood test laboratory biomarkers, such as neutrophil-lymphocyte ratio (NLR) and platelet-lymphocyte ratio (PLR), initially identified as prognostic markers in cancer^183^ and sepsis, show promise as systematic response markers following SCI. Elevated NLR has been correlated with increased respiratory infection incidence and reduced AIS conversion,^184,185^ while neutrophil percentage-to-albumin ratio (NPAR) indicates a reduction in the important plasma free radical buffering of albumin.^186^ Acute Respiratory Failure (ARF) is a severe complication of acute cervical SCI associated with high mortality. A predictive nomogram including admission NPAR, PLR, hemoglobin level, AIS, and NLI above or below C4 predicted the risk of ARF,^187^ although models incorporating these markers require broader validation. Lymphocyte markers are associated with post-SCI immune depression syndrome.

### Bioinformatics biomarkers

The serum and CSF biomarkers discussed above have primarily been related to injury magnitude and correlated to AIS and ISNCSCI outcomes. Bioinformatics approaches differ by applying much more comprehensive assays to uncover molecular signaling networks.

### Genetic variants as biomarkers

Genetic differences, such as single nucleotide polymorphisms (SNPs), contribute to variability in outcomes for patients with similar baseline neurological exams. Genomic analysis has uncovered polymorphisms relevant to SCI recovery variability, including variations in Brain-derived neurotrophic factor (Val66Met)^188^ and glial cell line-derived neurotrophic factor, cytokines (IL-6), and neurotransmitter receptors.^189^ An allele of the Apo-E gene (APOE*ɛ4)^190^ is associated with less recovery (Supplementary Table S3)^191,192^

### Proteomics biomarkers

Proteins are molecular engines of life processes such as DNA translation and oxygen transfer. Protein expression varies significantly among different cell types, and post-translational modifications, such as phosphorylation, modify protein function. Proteins interact in signaling complexes and control the functioning of the cell membrane and gene expression.

Systems medicine tools to understand the interaction and signaling between proteins are based on collecting, verifying, and integrating thousands of experiments within open-source molecular interaction network analysis platforms, such as Cytoscape.^193^ Ingenuity Pathway Analysis tools such as Causal Network Analysis (QIAGEN) provide context for omics dataset analysis, allowing researchers to identify upstream proteins interacting with the targets of interest. Clustering algorithms based on the “guilt-by-association” principle infer protein-protein interactions.^194^ Proteomics assessments have been used to compare human and research animal SCI responses to establish their mechanistic basis for pre-clinical validity. In a proteomics assessment comparing injured tissue from the rodent SCI model to acute human injury CSF,^10^ three common primary modules were identified: neural death, metabolic dysfunction, and cell growth and aging.^10^ Another proteomics study compared CSF and serum between human and experimental porcine SCI. Although the earliest time-points post-injury were similar, several human responses occurred later than in pigs. In both species, GFAP elevation was associated with injury severity and neurological outcome.^195^ Proteomics, like other omics analyses, requires robust methods for false positive detection due to multiple testing.

### MicroRNA biomarkers

MicroRNAs are short non-coding RNAs that regulate gene expression^196^ by binding to mRNA^168^ and inhibiting protein synthesis. They are transported by exosomes in the systemic circulation,^197^ and regulate immune function, inflammation, regeneration, cell death, neuroplasticity, motor recovery, and pain responses^198,199^ following SCI. miR-146a, one of the most abundant CNS miRNAs, is associated with several diseases due to polymorphisms that affect its functionality.^200^ In experimental SCI, miR-96 had neuroprotective effects, promoted cell proliferation, and reduced inflammation and apoptosis.^167^ miR-21 has been widely studied and found to affect multiple organ systems with roles in inflammation, cell proliferation, and apoptosis. Its dysregulation has been implicated in cancer, heart disease, and autoimmune disorders. In experimental SCI, miR-21 reduced cell apoptosis by decreasing the expression of critical genes such as PTEN and Fas ligand.^201^ Patients with degenerative cervical myelopathy have been observed to have elevated plasma levels of miR-21.^202^ Moreover, in a miR-21 knockout mouse model of cervical myelopathy, there was a notable reduction in microglial activation.^202^ In experimental SCI, miR-96 had neuroprotective effects, promoted cell proliferation, and reduced inflammation and apoptosis.^167^ In chronic SCI patients with neuropathic pain, there was a significant reduction in serum levels of miR-338-5p known to downregulate NMDA receptor signaling,^203^ and exosomes were found to contain miRNAs associated with accelerated vascular inflammatory disease in another study.^204^

### Limitations of body fluid biomarkers

Many markers obtained from serum and CSF after injury are recovered outside of their functional tissue-level context, which may leave their mechanistic relevance unclear. Examples include GFAP and NF, which reflect structural contents released from damaged cells without apparent signaling functions. Likewise, the tissue sources of genetic signaling molecules like miRNA or inflammatory cytokines may be unknown. Limitations like specificity, dynamic changes, and assay standardization must be addressed to better understand fluid biomarkers' role in injury responses and improve diagnostic and therapeutic strategies.

### Limited prediction of secondary conditions

SCI is thus characterized by sudden onset, gradual recovery, and evolution into heterogeneous chronic states. The extent of neurological recovery is considered the most critical outcome for the long-term outlook of an SCI patient. Most SCI recovery measure descriptors are heavily weighted to performance, such as operating a wheelchair or walking capability.^23^ However, given that SCI evolves into a chronic multi-system disease, other aspects of health and resilience are critical. Thus, the relevance of clinical assessments changes during injury evolution. The ISNCSCI can be used as both a predictor-independent variable and a dependent longitudinal outcome measure. Still, as SCI evolves, secondary states such as neuropathic pain can become more significant to the affected person than small differences in neurological scores. There are likely critical transitions between states that occur after the injury that underlie the establishment of secondary conditions such as neuropathic pain, metabolic syndrome, and dysautonomia.

### Correlations without mechanism

Currently, few clinical outcome measures for SCI are based on causal molecular mechanisms. Biomarkers have been evaluated in relation to the ASIA/ISNCSCI classifications using regression methods, but thresholds are adjusted to fit ordinal categories lacking mechanistic context. Retrospective correlation methodology is restricted to the data classes within the records. Machine learning (ML) techniques can potentially uncover previously unidentified correlations and latent relationships within datasets.^205^

### Mechanistically grounded variables and outcome measures

Predictors that connect mechanisms to end-points can have a powerful role in therapeutics development. In cystic fibrosis (CF), tests measuring sweat production identified mechanistically significant biomarkers, leading to the discovery of the CF transmembrane conductance regulator gene.^206^ Analysis of the sweat proteome revealed mechanistically related abnormalities in protein function.^207^ These assays have been instrumental in developing corrective drugs for CF.^208^ Neuropathic pain^209^ and spasticity^210^ are altered neurophysiological states amenable to Physiome/neural network modeling and systems biology network analysis that may be starting points to build up a more comprehensive understanding of phenotype evolution in chronic SCI.^211,212^

### Preserved spinal cord tissue

The extent of tissue preservation at the injury site is closely associated with recovery potential, a core concept in the SCI field.^213^ This premise forms the basis for neuroprotective and regenerative strategies. Clinical outcome assessments in persons with SCI have been correlated with the width of preserved tissue bridges on MRI.^148,214^ The number of preserved axons is considered a meaningful predictor of recovery and therapeutic effects.^215^ However, this relationship is often weaker than expected, resulting in a structure-function paradox.^216^ Over the past decade, epidural and transcutaneous electrical stimulation has revealed that connections supporting voluntary function may exist with minimal preserved tissue.^217^

### Neurophysiology

While the number of axons can be quantified in experiments, it may not directly correspond to function. Evoked potentials, including motor and sensory evoked potentials, are clinically accessible biomarkers to assess preserved connections following SCI.^218^ A transcranial motor evoked potential (MEP) conclusively demonstrates a “functional” connection, with MEPs correlated to the extent of preserved tissue bridges.^149^ When a therapeutic targets a specific neurophysiological mechanism, such as an ion channel, changes in evoked potentials can serve as a mechanistic outcome measure.^219^

## Integration of Bioinformatics, Biomarkers, and Clinical Data

Clinical measures such as the perceived severity of pain cannot foreseeably be replaced by entirely molecular methods. For bioinformatics data to significantly advance clinical insight in SCI, it needs to be integrated with existing clinical classifications and metrics. This requires several practical steps, including data curation, model development and validation, and testing in clinical practice.

Bioinformatics has informed how chronic multi-system disorders, such as diabetes and rheumatoid arthritis, progress through intracellular, intercellular, and organ interactions.^220^ Disease-informing mechanistic insights include genomic transcription, protein ensemble functions, and systemic signaling via cellular, endocrine, and exosomal systems. Emerging biological paradigms such as the miRNA interactome add mechanistic resolution to understand disease progression and therapy responses.^202,221^ Multi-system mechanistic biomarker models have been developed to move beyond a single point of therapeutic focus, such as the site of spinal cord damage, to integrate distributed multi-organ perturbations.^222^ By combining genetic bioinformatics data with health information, researchers can gain insight into how networks of altered gene expression are linked to disease characteristics.^221^ Clustering and topological analysis help to identify the most significant linkages, nodes, and critical variables.^223-225^ By identifying the most important nodes driving disease evolution, new customized treatment targets can be established.^61,226^

### Challenges related to classification language

Harmonizing medical information is essential for systems medicine to integrate potentially valuable but fragmented, variably defined, and differently formatted data. Core principles include data item standardization, quality assurance, privacy and security, and interoperability.

The World Health Organization developed the International Classification of Functioning, Disability, and Health (ICF)^96^ and the International Classification of Diseases (ICD) to establish standardized frameworks for global health communication. The ICF classifies health-related states into Body Functions and Structures, Activities, and Participation, providing a unified standard language and framework. The ICD and ICF mutually define disease components and their impacts on an individual's abilities. Standardized classification systems enable effective communication among healthcare providers, researchers, and policymakers, facilitating the development of tailored evidence-based interventions for individuals with specific health-related conditions.

### ICD, phenotype, and mechanisms

ICD is structured to provide a clear, unambiguous tree-structured classification system. For instance, ICD-11^227^ (ND51.2) defines spinal cord injury with a qualifier (8B4Y) indicating a non-traumatic SCI cause. Using ICD-11, a person with traumatic SCI can be characterized by a cluster of secondary conditions such as chronic central neuropathic pain (MG30.50), neurogenic bladder (GC01.4), and spasticity (MB 50.1). ICD's terminal branch parsing structure organizes diseases into static subcategories useful for diagnostic purposes but counterproductive for developing a multi-systems framework. Zhou and colleagues^7^ proposed revising ICD (as New Classification of Diseases [NCD]) to align the phenotypic ICD tree structure with critical molecular processes. However, ICD disease boundaries were found to separate pathophysiologic mechanisms that were similar between diseases. Paralysis, autonomic dysfunction, neuropathic pain, and spasticity are pathologies associated with SCI that cross disease boundaries. Multiple sclerosis and chronic SCI share neuroinflammatory mechanisms.^29^

The study of mechanisms influencing clinical phenotypes in SCI models necessitates terminology methods to integrate molecular details and conventional clinical data. The Systems Biology Markup Language (SBML) enables interoperability of biological process data for computational models^228^ and multi-scale physiomes^229^ using extensible markup language (XML) that is both human and machine-readable to define biological models mathematically. Importantly, ICD-10 codes are available in XML format. An illustration of using SBML for a Physiome-like in silico Reactome pathway is Biomodels Database Model:BIOMD0000000582, which displays cellular aging's impact on mitochondrial function (Supplementary Fig. S2).^230^

A current limitation is that SCI is not categorized as a “disease” in the Human Disease Ontology dataset, formatted in Web Ontology Language format (OWL) derived from XML, which integrates ICD and other classifiers. It does not include “spinal cord injury,” but instead includes several forms of “myelopathy.” Therefore, developing a data language that recognizes SCI as a multi-phenotype syndrome is an essential step to support clinical and molecular data element harmonization.^231^

### Data elements

Interoperability is crucial for computational process automation and data exchange.^232^ Different data sources, including free text, lab test results, imaging, and bioinformatics, can be interconnected by metadata models. The Systematized Nomenclature of Medicine (SNOMED CT) classification methodology allows for an individualized computational syntax and extensive classifier terms.^233^ It evolved from pathology nomenclature as a standard for electronic health records (EHRs), facilitating data reuse and information retention even when indexed to other formats. As compared to ICD, which is mono-hierarchical, SNOMED CT is a poly-hierarchical coding system in which concepts may have multiple “parents,” and thus link related conditions. It adds granularity to spinal cord pathology indexing by including edema (SCTID 65605001), ischemia (SCTID 371029002), and demyelination (308634000). Natural language processing (NLP) artificial intelligence (AI) search methods can be configured to return text or DICOM information mapped to SNOMED CT terms, and can remove Personal Health Information (PHI) during the search.

Precise data definitions are crucial in research; common data elements (CDEs) enhance interoperability and statistical power by enabling data aggregation in assessing clinical treatment, during clinical trials,^234-236^ and in meta-analyses. CDEs allow diverse studies to use standardized measures developed according to global metadata classification methodology, ISO/IEC 11179, to define entities and their attributes. The CDE property term is the research question, such as “cause of SCI,” and the value domain includes a specified set of answers. The National Institute of Neurological Disorders and Stroke of the National Institute of Health (NINDS) defined CDEs for major neurological disorders, including SCI,^237,238^ following the FAIR principle—Findable, Accessible, Interoperable, and Reusable. Accurate data definitions are also important in conducting EHR searches, with the Observational Medical Outcomes Partnership standardizing CDEs across EHR systems.^239^ The NINDS SCI CDEs include demographics, medical history, medications, endocrine and metabolic function, imaging, electrodiagnostic, and laboratory testing.^238^ If suitable standardization is achieved, CDEs could be expanded to incorporate molecular profiling.^240^

### Measurement properties and statistical challenges

Accurate, statistically analyzable measurements are necessary to predict and characterize neurological recovery and function in SCI. A systems medicine approach does not aim to replace existing outcome measures, which are relevant and enable valid treatment comparisons. Instead, it seeks to integrate multi-system data with validated measures to create models of SCI phenotypic and mechanistic evolution using more complex data analytics to potentially reveal causal factors.

The methods used to test associations in prognostic or research contexts depend on the type and quantity of data variables available. The Consensus-based Standards for the Selection of Health Measurement Instruments (COSMIN) provide a framework to evaluate outcome measures in the key domains of reliability, validity, and responsiveness.^241^ Most existing SCI ordinal classification and recovery scales can describe, but not explain, complex and composite multi-mechanistic phenomena such as “spasticity.” The absence of quantitative numerical intervals in clinical scales may create statistical obstacles when integrating bioinformatic data elements. However, clinical scales are pragmatic and often derived through extensive clinical experience. It is of great interest to determine if their clinical utility correlates with underlying mechanisms. A study on Huntington's disease effectively addressed this integrative challenge by identifying genes associated with the specific phenotype and correlating their expression levels with a recognized ordinal classifier.^242^

Innovative refinements of SCI outcome measures to improve statistical properties included a revision of the Graded and Redefined Assessment of Strength, Sensibility, and Prehension (GRASSP) test based on Rasch analysis to reduce item redundancy and improve interval properties.^243^ The Spinal Cord Ability Ruler (SCAR) is a recently developed scale that attempts to linearize ordinal ISNCSCI motor and SCIM scores.^244^

### Evolution of statistical approaches to integrate omics and clinical data

Data sets are typically analyzed to identify relationships between dependent variables, such as motor score change, and independent variables, like time to surgery. While parametric model-based regression methods are common in clinical research,^245,246^ SCI research increasingly employs nonparametric methods, transitioning from linear regression to mixed models that allow for multiple intercepts and covariate control.

Parametric statistical methods require assumptions about the distribution of the study population to calculate the mapping function, mean, and standard deviation for comparisons. In contrast, nonparametric techniques do not require such assumptions. Linear regression is typically used for continuous data, while logistic, proportional hazard, and mixed effect regression are employed for categorical, survival, and longitudinal data, respectively. Parametric methods may, however, perform better than non-parametric methods in smaller data samples.

Parametric regression methods assume linear relationships between predictors and outcomes, potentially overlooking complex interactions and requiring special consideration for predictor collinearity. Pre-defined research parameters constrain the assessment of unmeasured factors' impacts. In a complex problem like SCI, a univariate regression analysis might show a strong correlation that is actually due to other factors, necessitating multi-variate analyses to control for additional contributing variables. In contrast, non-parametric regression models identify significant associations from the data without a predetermined model structure, necessitating more data but offering greater flexibility in handling complex, multi-dimensional data. These models are developed using training data sets, balancing overfitting and underfitting, mitigating selection biases, and preserving input variable uniqueness. Non-parametric techniques may also aid in imputing missing values.^247^

### Dimensionality reduction

Omics data is characterized by large numbers of variables, as its use in medicine grows, it is essential to reduce data dimensionality to identify key variables that explain the greatest amount of observed variance in selected outcomes. Dimensionality reduction methods preserve explanatory power while reducing features. Principal Component Analysis (PCA) is often used for complex multi-variate analyses. By separating co-correlated groups of variables, PCA manages collinearity and helps identify the most relevant factors. For instance, PCA was used to determine the inflammatory cytokines most highly correlated with limb weakness in ICU patients.^248^

While PCA is a valuable dimensionality reduction technique, newer methods like independent component analysis, t-distributed Stochastic Neighbor Embedding (t-SNE), and non-negative matrix factorization offer benefits for specific applications in discovering gene expression modules.

### Machine learning

The large clinical data sets increasingly used to develop explanatory models for medical problems exceed human analytic throughput capacity, necessitating efficient computer-based approaches such as machine learning (ML). ML can improve the predictive accuracy of software applications without explicit programming. ML algorithms automate pattern recognition,^249,250^ predicting novel prognostic clusters by combining genetic, clinical, and lifestyle variables.^251^ The success of ML algorithms depends on proper labeling, aggregation, and organization. Unsupervised ML can employ neural networks, deep learning, and artificial intelligence (AI).

Of importance, ML does not replace statistical inference because its objective is prediction, not causal explanation. Also, ML-based decision support technologies are biased by the patient attributes used to construct the models, meaning they perform best on patients similar to those used in model development. The k-nearest neighbor and support vector machine approaches are a prominent nonparametric classification and regression method applicable to both systems biology^252^ and clinical datasets.

An example of ML capabilities is the use of the U.K. Biobank, a repository of biological and medical data, including Genome-Wide Association Study (GWAS), from a cohort of 500,000 people. Using a combination of clinical, lifestyle, and genetic data, researchers used archival MRI data to distinguish between individuals with and without non-alcoholic fatty liver and those with and without evidence of CVD. ML methods such as naïve Bayes were used to construct a prediction model, resulting in a regression tree of specific risk factor thresholds for the development of severe CVD.^251^ Similar modeling has been used to predict Alzheimer's disease progression.^253^ Using NLP on unstructured data, deep learning can identify distinguishing variables to improve decision-making in radiology.^254^

However, caution is necessary when using ML and AI, as sampling bias, data collection methods, and statistical approaches can all impact the algorithms and perpetuate errors and inequality. Iterative cycling may improve model performance as more data is incorporated. Reverse causality and confounding must also be considered when working with observational datasets.^255^

### Data integration between EHR and curated prospective datasets

ML can analyze data and identify new correlations, although substantial validation is necessary.^256^ EHRs are a vast source of clinical data that ML can utilize to answer research and clinical questions.^257^ Effectively integrated EHRs could also improve resolution in acute to chronic SCI data gaps caused by transitions from acute care to rehabilitation hospitals, and between in-patient to intermittent out-patients encounters. Despite the potential of ML to integrate large quantities of clinical, laboratory, textual, and imaging findings from patients with SCI, reliable anchoring classifiers are essential when dealing with error-prone assessments such as core neurological examinations. The curated prospective longitudinal NACTN registry dataset^258^ can serve as an anchor for ML nonparametric models in SCI. Data, including comorbidities, laboratory results, and patients' medications, can be sourced from EHRs alongside unstructured free text data from MRI, surgery, intensive care, and medical records. This data could be merged with existing datasets to develop decision-support tools. Recently, data from the COVID-19 pandemic have been incorporated into several critical care algorithms.^259^ Challenges include that clinical data collection and storage techniques vary, reflecting institutional policies and operational procedures. EHR NLP methods must be standardized and harmonized to ensure compatibility and consistency across different EHR sources.

### Unbiased recursive partitioning to create prognostic SCI models

SCI is a condition affected by multiple sources of heterogeneity. Stratification methods are an approach to create subgroups within data sets to reduce classification heterogeneity. One of the simplest stratification methods is by AIS subgroup. In a comprehensive multi-variate analysis using stratification, early infection was found to reduce voluntary voiding recovery at one year in individuals with AIS A-C, but not AIS D.^260^

Nonparametric regression utilizing recursive partitioning is a type of supervised ML stratification to parse large SCI datasets using combinations of existing classifiers. The unbiased recursive partitioning regression method (URP-CTREE) has used AIS, ISNCSCI, and other data (molecular/imaging) to improve neurological prognostication by reducing group heterogeneity. In this process, predictors related to the intended outcome are first identified, and all potential predictor pairings are recursively analyzed until a two-sample linear statistic discerns the two most distinct subgroups.^261^ This technique incrementally creates homogeneous dichotomous groups from initially heterogeneous groups based on model inputs and response variables.^261,262^ This method mitigates the limitations of AIS category breadth and provides more precise recovery prediction by identifying refined homogenous groups with similar outcome trajectories.^263,264^

However, unmeasured confounding variables can affect nonparametric recursive partitioning and large datasets are required to uncover confounding factors in apparently homogeneous groups.^265^ Multiple recursive testing necessitates specialized false positive correction and bootstrapping the original dataset to assess model overfitting. URP-CTREE has outperformed conventional linear regression in cervical SCI outcome prediction^261^ and has been used to generate a pneumonia decision support tool for SCI^266^ and to predict walking outcomes based on standard blood chemistry values post-SCI.^267^

### Integrated modeling of clinical and bioinformatics data

Bioinformatics contrasts with ISNCSCI, SCIM, and other refined clinical scales by using large amounts of high-resolution data with variable relevance, requiring effective organization methods. Syndromic SCI classification might better align clinical data clusters and molecular networks related to specific pathologies like neuropathic pain and metabolic syndrome. Identifying prominent molecular changes necessitates comparison data from uninjured individuals and those with SCI, with and without the syndrome of interest. Depending on the biological sample sources, it might be feasible to construct datasets representing inflammatory, immune, and metabolic signaling to detect the most or least shared markers between phenotypes. Changes in molecular modules can be cross-checked against measured allostatic load and epigenetic markers, and deviations from normative data might correspond to increased allostatic load. Pathway Integromics in Cancer^268^ has been used to synthesize bioinformatics and clinical data into predictive and interpretive models.

Since most omics provide single time-point snapshots, multiple sampling is needed to elucidate temporal changes, identify non-linear properties,^269^ and critical transitions. The high granularity of omics analyses may obscure emergent factors,^270^ such as resilience.^271^

### Gene expression and transcriptional regulation in SCI

SCI and recovery are linked to changes in the expression of hundreds of genes across several tissues and organs. Transcriptomic data may be examined using differential gene expression (DEG) and unsupervised hierarchical clustering techniques. Adequate control data sets are essential for DEG^272^ to identify genes with differing expression between SCI and control states, then hierarchical clustering to discern if the differentially expressed genes belong to meaningful clusters indicating co-regulation or a biological pathway. In acute SCI, downregulated genes related to neuronal function pathways, while upregulated genes supported inflammatory and immunological responses. Transcriptomics analyses of the limited regeneration after SCI have identified several molecular cascades controlled by “master” regulators,^273^ including mitogen-activated protein kinase (MAPK),^274^ ATF3,^275^ and PTEN.^276^ DEG studies have identified that MAPK and Ccl3, a macrophage inflammatory cytokine systemically transported in exosomes, are associated with the emergence of neuropathic pain.^277^ In the TRACK-SCI program, whole blood cells from acute SCI patients underwent unsupervised co-expression network analysis. DEG and gene expression modules were correlated to AIS grade,^278^ and differences in immune cell modulation were observed between AIS A versus AIS D patients.^278^

### Network analysis

Omics data require network analysis tools to interpret correlations, given the high biological complexity and potential for non-linear and stochastic interactions between variables. These tools include gene-gene co-expression, transcription factor regulatory networks, protein-protein interactions, and signal transduction networks. When comparing control and injury datasets, the null hypothesis of no difference is repeatedly tested, and data is ranked by effect size and most significant *p* values. Due to many comparisons, the false positive discovery rate must be controlled using methods like the Benjamini-Hochberg procedure.

The topology of clinical phenotypes and their molecular and physiological substructure may be deduced using clustering coefficients and centrality measures using tools like MCODE in Cytoscape.^279^ Topological transcriptomic network analysis identifies probabilistic networks that consist of nodes showing potentially causal relationships between variables, with connected edges representing gene expression facilitation or inhibition (Fig. 5). The path length between nodes may indicate the number of signaling or genetic regulatory steps between nodes. Unconnected nodes are considered independent variables under the data acquisition conditions.^280^ Sample size, effect size, and false discovery rate influence the analysis, and some information may be lost during feature extraction and dimensionality reduction.

## Modeling of SCI as a Multi-System Disease

For decades, researchers have focused on the spinal cord injury site, where the loss of neural integration and normal inhibition is followed by neuroplastic reorganization that extends to organ system abnormalities.^281^ Although much research has focused on the recovery of motor systems, a systems approach is naturally suited to the extensive distribution of the autonomic nervous system (ANS).^282,283^ Post-SCI pathological syndromes interact in complex ways, that is, gut dysbiosis worsens systemic inflammation,^221^ which in turn can accelerate CVD.^284^ An interactive systems model may offer greater explanatory power, enabling predictions not only for neurological prognosis but also for estimating vulnerability and resilience.^285^ Multi-system involvement in SCI has implications for acute care, complication reduction, recovery-promoting therapies, and life with chronic SCI. Other multi-system diseases, including diabetes, have undergone extensive modeling.

### A systems biology approach to SCI in subacute and chronic stages

A systems biology framework can be both explanatory and prognostic, an important distinction.^286^ We propose using clinical phenotype clusters (e.g., AIS B-severe neuropathic pain-prominent dysautonomia) to serve as anchors for molecular network assessments, critical transition events, and the generation of Physiome models. We suggest an initial approach to SCI systems-medicine via multi-variate modeling casting a wide variable net across different scales,^287^ including demographics factors (age/sex), AIS, MRI injury severity, multi-organ injury, serum biomarkers, genetic characterization (GWAS) to identify relevant SNPs, peripheral blood inflammatory response and immune depression markers, autonomic instability and blood pressure, complications, and microbiome changes.^288^ Serial measures are important to understand how phenotypes such as neuropathic pain and metabolic syndrome evolve, the impact of allostatic stress, and to assess epigenetic markers, including aging.

#### Accessible specimens for systems biology

Omics analysis can be performed on accessible specimens, including serum, circulating blood cells, microbiome samples,^289^ and limited tissue biopsies such as muscle. Peripheral blood mononuclear cells provide information pertinent to inflammation, immunity, and metabolism after SCI.^290,291^

Notable phenotype-bioinformatics studies include using Systems Biology Cohorts of Veterans and active-duty military personnel, investigating molecular differences between those with and without diagnosed post-traumatic stress disorder (PTSD).^292^ In this study, the phenotype condition was dichotomized (has/has not) and compared with blood cell–derived genetic, epigenetic (methylation), transcriptomic, miRNA, proteomic, and metabolomic assay data. Topological patterns across the data were assessed using multiple tools, including weighted gene co-expression analysis and Consensus Topological Overlap. Notable findings included heightened inflammatory responses to stress, high levels of protein phosphorylation, and reduced neuron projection markers. Notably, PTSD has been linked to allostatic load and mitochondrial dysfunction.^293^

Some clinical studies have found correlations between proteomic patterns and neuropathic pain.^294^ It is believed that several forms of pain sensitization disorders share mechanisms. Complex regional pain syndrome-associated genes have been identified through RNA transcriptomics from the blood of affected patients,^295^ revealing epigenetic DNA methylation changes in inflammation and immune-regulating genes.^38^ In the Veterans Integrated Pain Evaluation Research study (VIPER),^296^ amputees with phantom pain were dichotomized based on pain scores greater or lesser than 3/10, and their blood samples were analyzed using an extensive neuroinflammation panel. The model also assessed pain catastrophizing, which might be considered an analog of reduced resilience. The two independent categorical groups in the cross-sectional study were compared using a Mann-Whitney non-parametric U test with catastrophizing modeled as an indirect effect.^297^ A validating study cohort was created employing the BioVu DNA biobank; the deidentified GWAS dataset was used to examine the correlation between epigenetic changes and ICD-9 pain phenotype diagnostic codes found in the Synthetic derivative, a Vanderbilt de-identified EHR database, providing a control group of 20,000 records.^38^ A further study using BioVu examined for shared polygene profiles across multiple pain types with combinations of SNPs assessed against EHR ICD classification pain data using PCA.^298^

### The autonomic nervous system multi-organ integrative system

The ANS regulates homeostatic processes such as immune function through the sympathetic-adrenal medullary axis, hypothalamus-pituitary brainstem axis, and parasympathetic systems.^299,300^ Post-SCI ANS abnormalities may be primarily related to neural axis injury level or injury completeness.^36,301^ Initial post-injury neurogenic shock, caused by loss of sympathetic tone, predicts poor neurological recovery.^6^ Severe cervical SCI ANS disruption leads to immune dysfunction through splenic atrophy,^302^ altered metabolism,^303^ and susceptibility to septic shock.^304^

Injury above T6 is a crucial threshold for disturbed autonomic regulation due to the loss of control over the splanchnic vascular bed. Chronic post-SCI blood pressure instability is more common than previously understood.^305,306^ Autonomic dysregulation leads to the uncontrolled release of catecholamines and glucocorticoids, impairing immune function.^307^ Autonomic dysreflexia (AD) occurs when a normal sensory stimulus inappropriately results in severe hypertension. AD can depress the immune system, increasing infection risk by reducing splenic leucocytes and increasing glucocorticoid release.^32,308^ Indicators of ANS injury severity include low serum norepinephrine and low-frequency systolic blood pressure fluctuations.^309^ Increased allostatic load and autonomic dysfunction have been linked to several neurodegenerative diseases.^310^

### Quantification of allostatic load

Allostatic load (AL), a measure of stress-induced physiological and somatic damage accumulated over the lifespan”^311^ may inform the SCI systems medicine-Physiome approach^52^ and provide therapeutic targets. Allostatic load indexes (ALI) include physiological and serum biomarkers of inflammation, neuroendocrine, metabolism, cardiovascular health, and body mass index.^312^ Originally, ALI assigned a score of 1 for each composite measure outside a population-specific biomarker upper limit, yielding a score from 0-8.^313^ The low-frequency component of heart rate variability (HRV), impaired by sympathetic disruption, is also considered a quantitative measure of allostatic load.^309,314,315^ Epigenetic changes, including CpG methylation, indicative of biological age,^316^ have been correlated to allostatic stress measures.^317^

AL has been linked to hypercortisolemia, gut microbiota dysbiosis, elevated proinflammatory cytokines, decreased synaptic plasticity, and hypothalamic-pituitary-adrenal (HPA) axis disruption.^318^ In individuals with SCI, conventional AL indexes may require modification due to cardiovascular and HPA axis disruption,^52,319^ with metabolic, neuroendocrine, cardiovascular, and immunological markers proposed for inclusion in an allostatic burden index.^52^

Mitochondrial performance, impaired by AL, is a major physiological and molecular contributor to health. Its dysfunction impairs cellular metabolism, generates toxic free radicals, and induces apoptosis.^56^ In a cross-sectional study, markers of metabolic syndrome, such as increased visceral adipose tissue, elevated IL-6, and CRP,^320^ and low testosterone, predicted mitochondrial dysfunction.^321^ Additionally, adrenal dysfunction can increase cortisol, norepinephrine, and glucose levels, triggering mitochondrial fragmentation.^322,323^

Sleep dysfunction and circadian variations increase allostatic stress.^324,325^ Variations in the microbiome can mitigate or worsen AL,^318,326^ and dietary factors can modify oxidative stress through important signaling modules such as NF-Kappa B.^327^ AL is also associated with external factors such as low socioeconomic status.^328^ Factor analysis could be used to assess SCI-specific allostatic stress indicators to identify the most contributory parameters.^329^

### Emergent conditions of relevance to spinal cord injury systems biology

#### Resilience and frailty

Resilience can mitigate allostatic stress. Resilience, a multi-dimensional concept, is an essential determinant of outcomes after SCI.^330^ Biologically, resilience represents the system's capacity to return to its baseline state after stress, thus mitigating AL by lowering the response magnitude to stressors. It shares biological markers with AL indices, including cortisol, HRV, and immune cell reactivity. Additionally, resilience is enhanced by certain 5-HT gene polymorphisms.^51,331^ Its opposite, frailty, is an emergent state resulting from dysregulation in multiple systems that predicts morbidity and mortality post-SCI.^54^ It is assessed through measures of homeostatic perturbation, such as the Mahalanobis distance, which serves as a multi-variate index for comparison to initial or average undiseased states.^332-334^

#### Chronic pain

Pain, as a stressor, is linked to AL^335^ and affects many individuals with SCI chronically, with multiple effects at the brain and physiological levels. Neuropathic pain is a recognized AL source correlated to biomarker predictors,^24^ including MRI ventral tissue preservation^336^ and thermal pain adaptation,^337^ and is mitigated by resilience.^338^

#### Metabolic syndrome

Metabolic syndrome is a pro-inflammatory source of allostatic stress and accelerated aging.^339^ SCI leads to muscle atrophy and diminished endocrine myokine upregulation^340^ after exercise.^341^ In chronic SCI, cross-talk between muscle and bone regulates their respective catabolism after SCI,^342^ and high fat-to-lean body mass ratios associate strongly with metabolic syndrome and systemic inflammation.^343^ Injury level–dependent leptin levels increase, and leptin resistance impairs satiety, aggravating obesity.^344^ Metabolic syndrome can also cause brain dysfunction due to abnormal glucose metabolism.^345^ The Virtual Metabolic Human Database is an example of a systems medicine resource that integrates microbiome metabolism with nutrition and disease.^346^

#### Aging

Accelerated aging, a multi-system emergent effect, can occur due to increased AL after SCI^328,347^ and be measured with epigenetic clocks.^316^ In individuals with MS, increased aging has been detected in neurons.^348^ A reliable indicator of physiological dysregulation and AL is the statistical distance of composite biomarkers from normative values.^349^ These models predict mortality, adaptive capacity, and allostatic burden^350^ and could be adapted to SCI.

#### Exercise as a multi-system treatment

Immobility leads to complications and increased comorbidities.^351^ Exercise, a multi-system therapy, mitigates allostatic stress post-SCI,^352^ with HRV as a response biomarker.^353^ Exercise can reduce neuropathic pain,^354^ mitigating abnormal transmembrane chloride.^355^ In uninjured individuals, exercise induces epigenetic changes in skeletal muscle, including microRNA changes.^356^ Even after electrical-stimulation-evoked muscle exercise in people with SCI, epigenetic methylation changes are detectable in multiple genes.^65^ In rodent experimental SCI, exercise reduced injury cavity size and increased DNA demethylation in the brain.^357^

### Assembling the SCI systems medicine model

#### Multi-phenotype classification

**FIG. 6.**
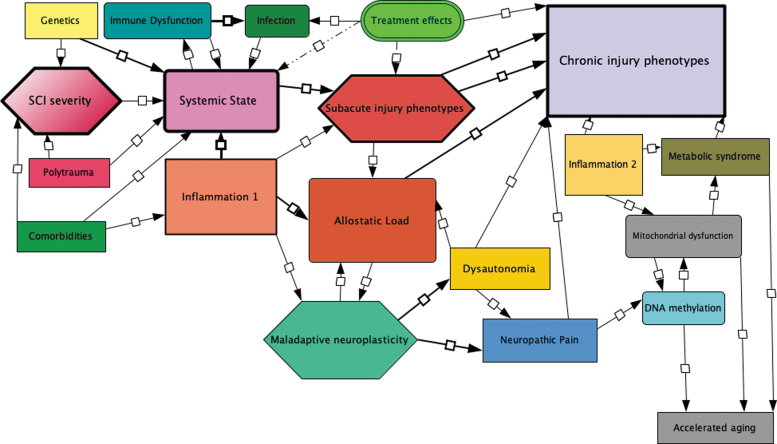
Network model. This is possible network model presented in a modular and interconnected systems medicine framework composed in Cell Designer Version 4.4.2. Key nodes are maladaptive neuroplasticity, mitochondrial dysfunction, dysautonomia, treatment effects, and chronic injury.

Systems biology modeling can be approached either through a granular bottom-up or simplified top-down approach.^358^ To develop a multi-systems SCI framework, we have considered the integration of bioinformatics, physiological, allostatic, and clinical data (Table 2; Supplementary Fig. S3). We propose classifying SCI using a syndromic model with secondary conditions and their severity as phenotypes (e.g., Fig. 6).

**Table 2.** Syndromic Formulation of Spinal Cord Injury: Data Layers

Demographics	Age, biological sex, time since injury
Covariates	Prior diabetes, cancer, mental illness
Clinical measures	AIS, ISNCSCI, SCIM, spasticity, autonomic standards, neuropathic pain, CDEs.
Clinical events	Infection, hypotension, surgical decompression, depression
Phenotypes	ICD codes, SNOMED CT
Predictive Biomarker	MRI, acute serum and CSF injury biomarkers if available.
Autonomic variables	SCI standards, HRV, orthostasis, dysreflexia
Drugs and treatments	E.g., Baclofen, gabapentin, antibiotics
Bioinformatics	Genetics. GWAS as available, miRNA, SNPs Inflammatory, immunologic, metabolomic, and microbiome markers
Allostatic load	SCI Allostatic load index, biological clock
Chronic phenotypes. Metabolic syndrome	Biomarkers, metabolic syndrome severity index/scale
Chronic phenotypes. Neuropathic pain	Pain scales, neuropathic features
Resilience measures	Exercise capacity and intensity, personal autonomy,

AIS, American Spinal Injury Association Impairment Scale; ISNCSCI, International Standards for Neurological Classification of Spinal Cord Injury; SCIM, Spinal Cord Independence Measure; CDE, common data element; MRI, magnetic resonance imaging; CSF, cerebrospinal fluid; HRV, heart rate variability; GWAS, Genome Wide Association Study; miRNA, microRNA; SNP, single nucleotide polymorphism; SCI, spinal cord injury.

Patient similarity networks could provide a framework to assess SCI phenotype heterogeneity and to identify patient subgroups. In this approach, patients are represented as nodes, and connections are established based on similarities. As a top-down approach, we propose to map individuals with SCI based on clinical phenotype-secondary condition clusters and subsequently test for correlations to bioinformatics data. Alternatively, a granular bottom-up approach is to perform unsupervised learning analysis on the patients integrated clinical and bioinformatic data to assess for related clusters. By examining the relationship between complication phenotypes and molecular clusters, we can detect critical nodes underlying phenotype evolution using network analysis.

#### ANS Physiome

SCI is a syndrome of multi-system perturbations with a spectrum of evolved secondary states affecting individuals. Although there are currently no SCI Physiome models, the ANS shows promise in understanding post-SCI states due to its broad multi-organ distribution, measurable variables such as blood pressure and HRV, known anatomical connections, and neurotransmitters. Developing an ANS Physiome could incorporate indices of allostatic stress, metabolic syndrome, chronic inflammation, and maladaptive immune responses.

The PINE model (Psycho-immune neuroendocrine Physiome) was designed as a systems medicine approach to major depressive disorder.^60,359^ It integrates the HPA axis, the ANS, and metabolic immune and inflammatory cytokine signaling.^359^ By incorporating key molecular interactions, the PINE model addresses disrupted homeostatic function caused by chronic stress, which generates AL and increased risk for major depression.

Creating an ANS Physiome requires model validation on different datasets, simulations with varied input parameters,^20^ and testing across diverse populations to ensure robust development. A successful ANS Physiome model would enable predicting potential therapeutic consequences through test inputs.

To integrate clinical phenotype, allostatic load data, and molecular data from the same patients, it is necessary to align and unify them in a standardized format. Clinical phenotype data is analyzed to identify clusters representing distinct patient subgroups. To incorporate molecular networks, allostatic load, and phenotypic clusters, module detection or community structure analysis is utilized. Statistical methods can then be applied to test the significance of associations and assess robustness using permutation testing or bootstrapping.

To create tables for ML, a series of layers will be combined to construct sets of data features (Table 2). This parameter matrix will be used in a stepwise data integration strategy^360^ to derive labels and their relationships to determine if multi-variate clusters (e.g., GWAS, inflammatory) align with individual phenotypes (e.g., complete injury with neuropathic pain, metabolic syndrome, and advanced biological age). In the process, the strength of the association of contributing variables to respective phenotypes may be deduced, and the main molecular drivers identified. Nodal interaction points between molecular changes and the Physiome models may be established, with AL as a unifying axis/concept.

#### Syndromic classification of SCI

The layers used to construct the initial data matrix are depicted in Table 2. The axes of allostatic load and resilience may be useful for mapping SCI syndromes (Fig. 6).

To progress towards this goal, we consider the following steps:

1. Conducting a patient similarity network analysis from adequate datasets to establish initial categories based on phenotype-secondary conditions, thereby identifying groups of patients with similar characteristics.
2. Encode phenotype clusters in language suitable for ML.
3. Perform network analyses to determine if consistent molecular network modules underly phenotype-secondary conditions.
4. Create initial systems Medicine Models for validation testing.
5. Refine the current measures of allostatic load to ensure their specificity to SCI.
6. Develop the SCI-ANS Physiome, a model to integrate SCI and autonomic alterations with clinical, allostatic load, and bioinformatics data at relevant time-points after SCI.

### Limitations of an allostatic load systems medicine-Physiome model

A comprehensive understanding of SCI at a systems level is important to therapeutic progress. However, the systems biology approach is at a very early stage, and models may be affected by several extrinsic factors that contribute to variation in outcomes after SCI. Examples of such factors include polypharmacy, the effects of antibiotics on inflammatory responses, and alterations in the intestinal microbiome.^361,362^ Additionally, sociodemographic factors contribute to allostatic stress and may be challenging to quantify accurately. Given the complexity of the project, it is prudent to start by assembling phenotype-molecular networks where substantial knowledge already exists, such as understanding linkages between neuropathic pain and inflammation.^277^

## Summary

Acute SCI is a critically dysregulated dynamic multi-system condition characterized by multiple interacting molecular and physiologic modules, some supporting recovery and others producing chronic multi-system dysfunction. Each patient's presentation and recovery trajectory are unique and influenced by multi-system, multi-injury, environmental, social, and psychological factors. Chronic SCI evolves through a multitude of altered physiological and molecular events. By far, motor function has been the axis on which recovery is measured, and in a general way, other systems are expected to correlate. Current SCI classifiers are mainly ordinal, predate the advent of bioinformatics, and lack biomolecular grounding. Current data elements assign definitions to multi-factorial and complex phenomena such as “neuropathic pain” and “spasticity.”

There are significant data gaps between the intense acute care period, the limited acute rehabilitative period, and the remainder of the individual's life with chronic SCI. Limitations of current classification and prediction measures are evident in the disparities in recovery from seemingly identical initial damage patterns. Clinical trials falter because group means-based statistical approaches are inherently limiting for a condition with so much individual variation. In addition to motor recovery, emphasis should be placed on the prevention of secondary conditions that, once established, are difficult to reverse.

SCI disrupts homeostasis, leading to secondary conditions that are established during the acute and subacute injury periods. Integrative functions of the ANS are lost to variable degrees. Allostatic stresses contribute to accelerated aging and perpetuate complications such as neuropathic pain, and eroding resilience. Although several injury biomarkers have been identified as prognostic biomarkers in SCI, many are descriptive and have not been developed into validated mechanistic or predictive models.

Considering the interactions among critical systems after SCI, a systems medicine approach can support models of individual change over time and identify critical transition states. However, the current multi-system modeling of SCI is in early stages. It is important to identify the most accessible systems and leverage existing resources by assembling multidisciplinary expertise.^6^

The NACTN registry and associated sub-studies document the acute to chronic injury phase, primarily in the first year, focusing on neurological recovery to inform natural history and the evidence basis to validate acute care practices. NACTN investigators are committed to enhancing recovery after SCI through advanced critical and surgical care and therapies to mitigate injury^138^ and potentiate neuroplastic and regenerative restoration.^363^ Attempting to understand the molecular and physiological basis of secondary conditions within an integrated framework has yet to be a research focus. For NACTN to develop a systems medicine focus; important steps would include the ability to access EHR data from associated acute care and rehabilitation hospitals. Collaborations would be needed to obtain and store genomics and biological samples, conduct omics analysis, and integrate the data into multi-variate data sets. Allostatic load measurement could be initiated, especially in the early post-injury period. If systems medicine approaches identify critical nodal thresholds, ML-based decision support tools could be derived to assist clinicians in optimizing decisions to reduce the incidence and severity of chronic SCI phenotypes. Understanding the temporal drivers of SCI syndrome states should lead to new and more effective therapies.

## Transparency, Rigor, and Reproducibility Summary

This review does not report primary data.

## Supplementary Material

Supplemental data

Supplemental data

Supplemental data

Supplemental data

Supplemental data

Supplemental data
