# Supplementary material for: Development of a Systems Medicine Approach to Spinal Cord Injury
Source: J Neurotrauma. 2023 Aug 23;40(17-18):1849–77. doi: 10.1089/neu.2023.0024 (PMC10460697; doi:10.1089/neu.2023.0024)
Supplement: Supplemental data [file Suppl_TableS1.docx]

**Supplemental Table 1. Established patient predictors of neurological and functional outcomes following SCI.**

| **Outcome type** | **How outcome is commonly measured** | **Associated with improved recovery** |
| --- | --- | --- |
| Neurological | ISNCSCI | Incomplete injury  Presence of zone of partial preservation  Younger age  More caudally located injury  Non-penetrating injury (vs penetrating)  Certain genetic polymorphisms |
| Functional | Ambulation  Functional independence measure  Spinal cord independence measure | Less severe baseline neurological exam  Fewer complications during hospitalization  Engagement in physical therapy during hospital stay  Younger age  Length of stay  Delayed discharge to rehab (-ve) |
| Mortality | Death | Greater severity of baseline exam  More cephalad level of injury  Concurrent additional injuries  Concurrent TBI  Pre-existing comorbidities  Age |
