# Supplementary figures and images for: Development of a Systems Medicine Approach to Spinal Cord Injury
Source: J Neurotrauma. 2023 Aug 23;40(17-18):1849–77. doi: 10.1089/neu.2023.0024 (PMC10460697; doi:10.1089/neu.2023.0024)

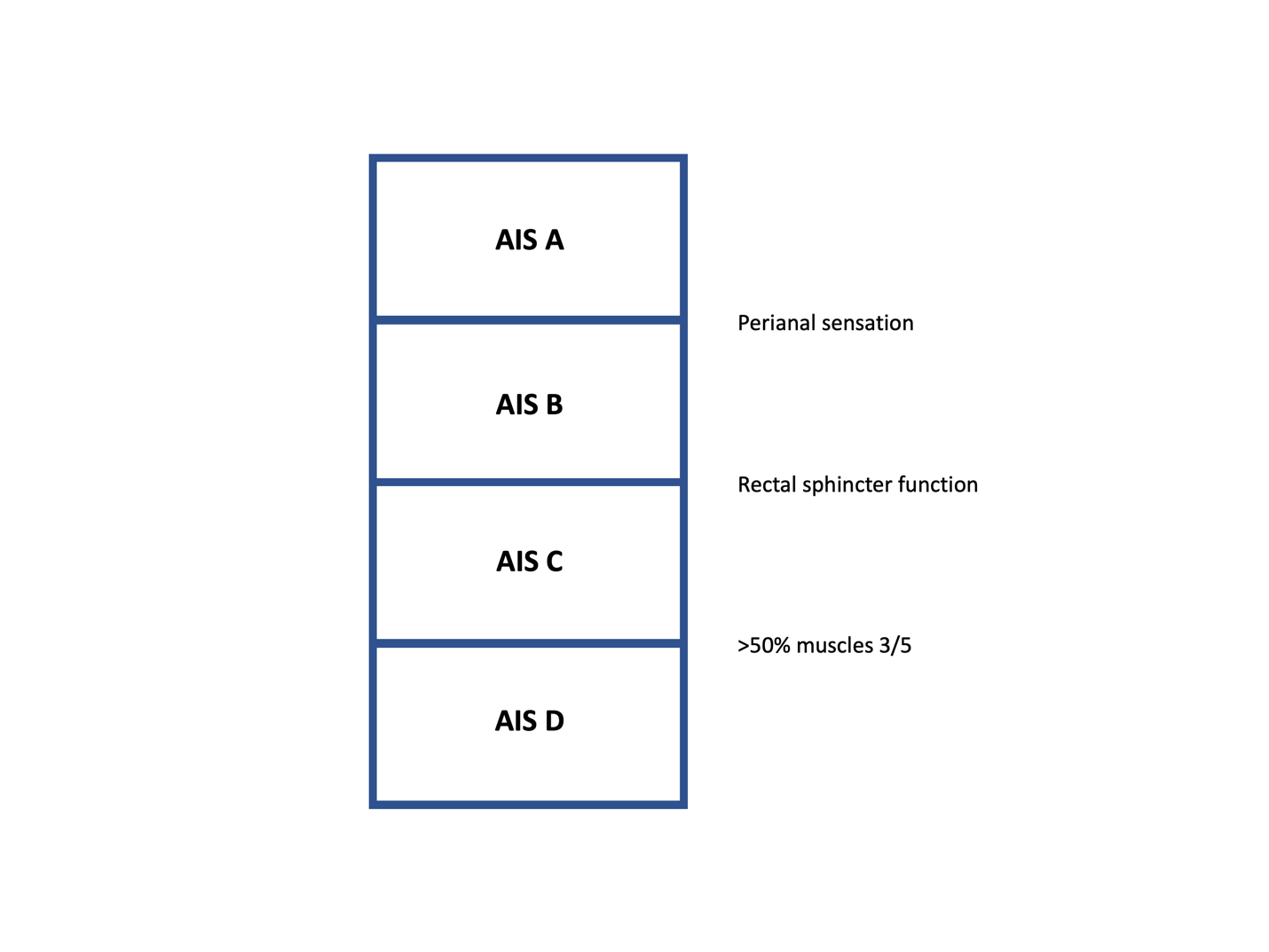


**Supplemental Figure 1**. Asia Impairment Scale.

Supplement: Supplemental data [file Suppl_FigureS1.docx]

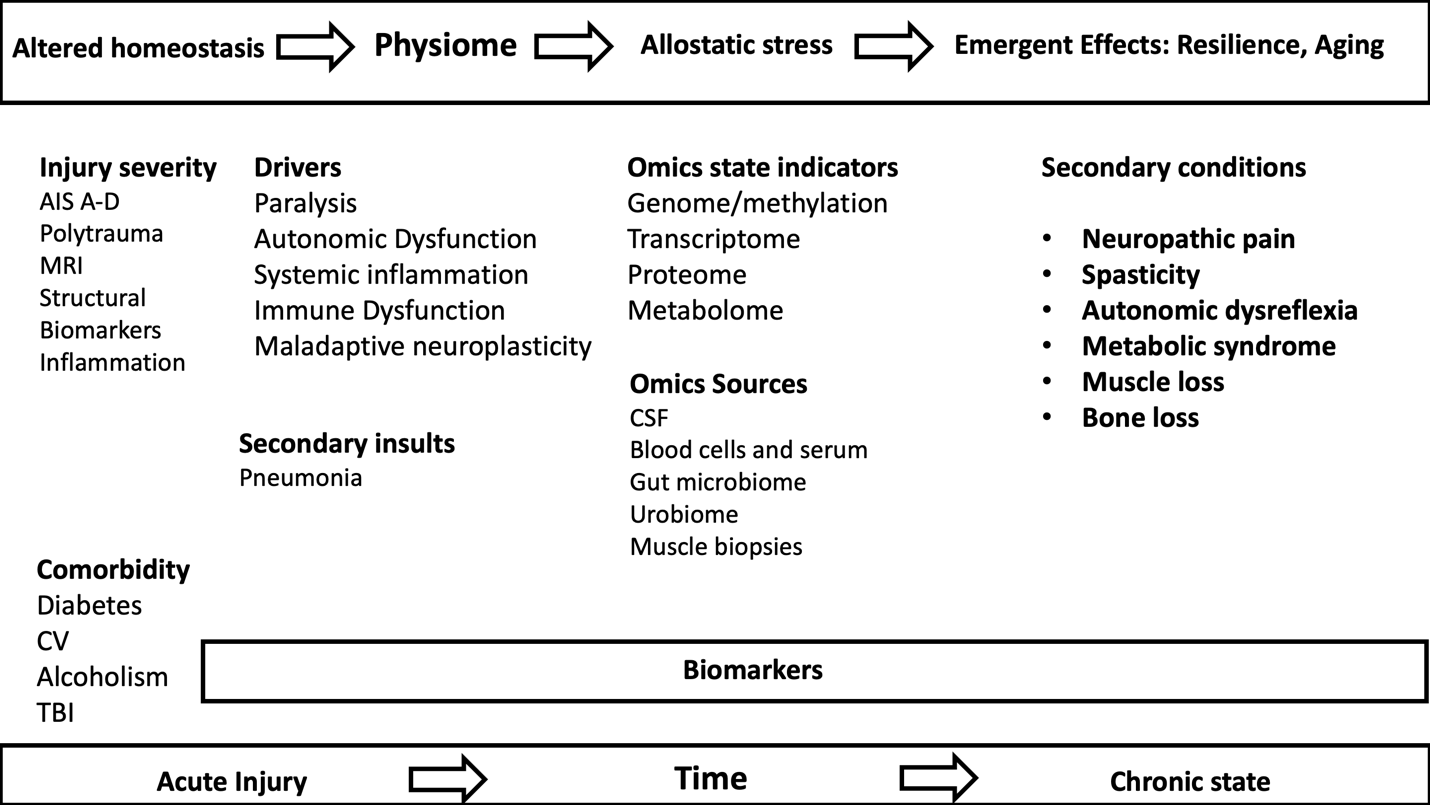


**Supplemental Figure 3.** Systems Biology Schematic

Supplement: Supplemental data [file Suppl_FigureS3.docx]
