# Supplementary material for: Development of a Systems Medicine Approach to Spinal Cord Injury
Source: J Neurotrauma. 2023 Aug 23;40(17-18):1849–77. doi: 10.1089/neu.2023.0024 (PMC10460697; doi:10.1089/neu.2023.0024)
Supplement: Supplemental data [file Suppl_TableS2.docx]

**Supplemental Table 2.** Established classification and prognosis of acute spinal cord injury using MRI.

| **Imaging feature** | **Association** |
| --- | --- |
| Spinal cord signal change on T2-weighted MRI | Presence of intrinsic signal change acutely after injury associated with greater risk of adverse events and less chance of ambulation at follow-up^151^ |
| The Brain and Spinal Injury Center (BASIC) MRI score | Scores correlate with neurological function at admission and are predictive of functioning at discharge^152^ |
| Diffusion tensor imaging (DTI) tractography | Indices obtained from a region of interest inferior to the injury epicenter correlate with total ISNCSCI score^155^ |
| Hematoma on MRI | Presence of intra-axial hematoma, length of hematoma, length of spinal cord edema, cord compression by extra-axial hematoma predictive of baseline and follow up neurological exams^153, 383, 384^ |
