# Supplementary material for: Development of a Systems Medicine Approach to Spinal Cord Injury
Source: J Neurotrauma. 2023 Aug 23;40(17-18):1849–77. doi: 10.1089/neu.2023.0024 (PMC10460697; doi:10.1089/neu.2023.0024)
Supplement: Supplemental data [file Suppl_TableS3.docx]

**Supplemental Table 3.** Emerging structural, response, microRNA, and inflammatory markers measured acutely-post injury associated with spinal cord injury prognosis.

|  | **Biomarker** | **Association** | **References** |
| --- | --- | --- | --- |
| **CSF** | | | |
| Structural | tau | -Correlation with baseline AIS score  -Predictive of AIS motor score improvement at 6 months | ^79^  ^159^ |
|  | glial fibrillary acidic protein |  |  |
|  | S100β |  |  |
| Inflammatory Response Cytokine | IL-6 | -Correlation with baseline AIS score  -Predictive of AIS conversion at 6 months | ^171^ |
|  | IL-8 |  |  |
|  | monocyte chemotactic protein-1 |  |  |
| **Serum** | | | |
| Structural | GFAP | Increased following SCI | ^172^ |
|  | NSE | Increased following SCI.  Higher levels associated with more severe SCI acutely | ^173^ |
|  | pNF-H |  |  |
|  | NF-L | Levels higher in complete SCI compared to incomplete or central cord syndrome. NF-L levels were predictive of motor outcomes up to 12 months post-injury. | ^165^ |
| Inflammatory | TNF-alpha | Higher levels correlated with neuropathic pain.  Lower levels correlated with improved AIS grades at 3 and 6 months | ^181^  ^177^ |
|  | IL-1β | Levels increased following SCI. |  |
|  | TGF-beta | Increased following SCI. | ^180^ |
|  | Inflammasome activation | NLRP-1 | ^385^ |
|  | sCD95L | Increased following SCI. Higher levels associated with less recovery |  |
|  | IGF-1 | Increased following SCI.  Higher levels associated both with less and greater chance of recovery | ^161^ |
|  | Neutrophil/lymphocyte ratio | Higher ratio predictive of acute complications (respiratory infection) and lower 6-month AIS grade in cervical traumatic SCI patients | ^184, 185^ |
| **Genes and genetic regulation markers** | | | |
|  | microRNA | Evidence still emerging, limited to animal models, miRNA-21 | ^167^ |
|  | Apo E, BDNF | Associated with neurological outcomes | ^386, 387^ |
