# Supplementary material for: Development of a Systems Medicine Approach to Spinal Cord Injury
Source: J Neurotrauma. 2023 Aug 23;40(17-18):1849–77. doi: 10.1089/neu.2023.0024 (PMC10460697; doi:10.1089/neu.2023.0024)
Supplement: Supplemental data [file Suppl_FigureS2.docx]

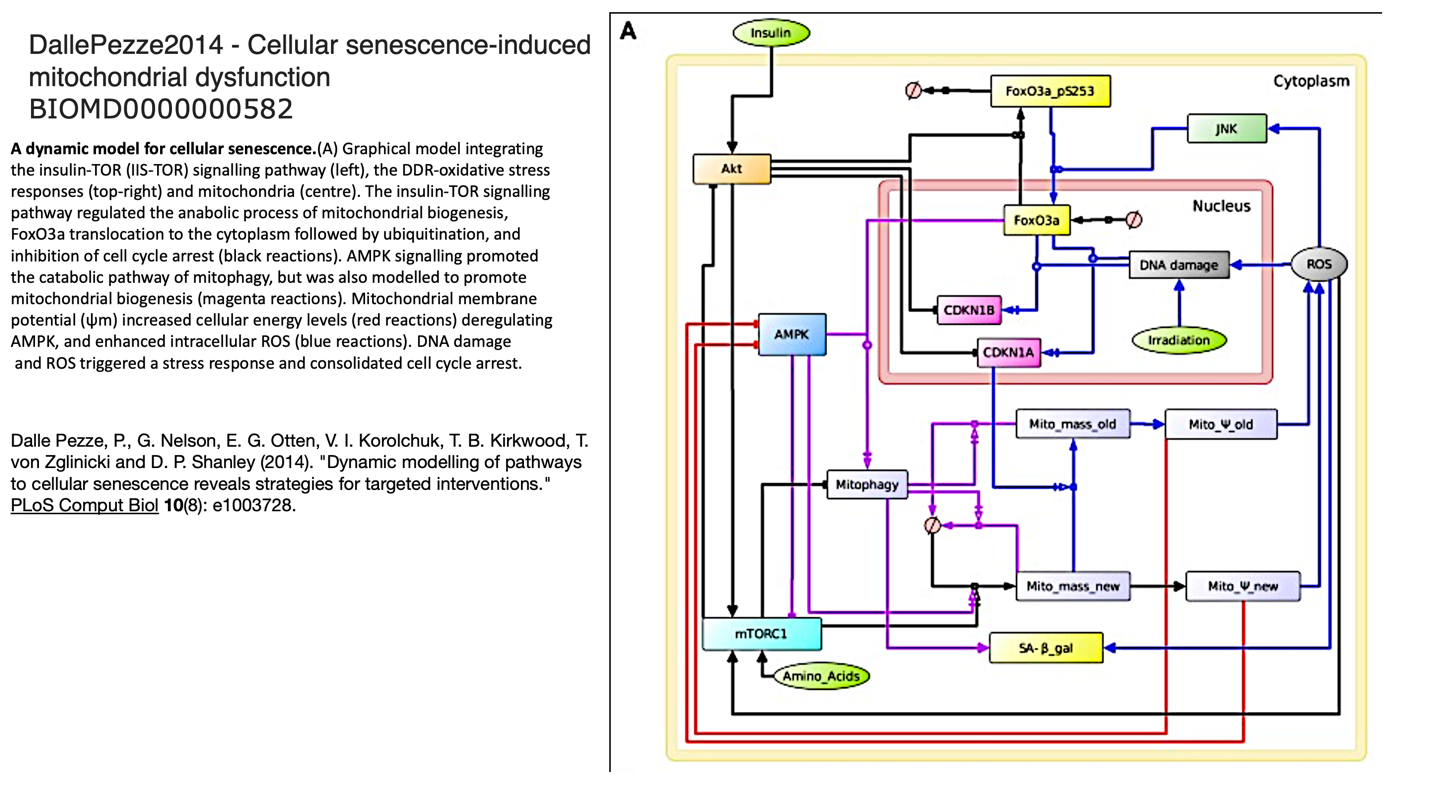


**Supplemental Figure 2.** A representative reactome coded in SBML. Open source see:

https://www.ebi.ac.uk/biomodels/BIOMD0000000582
